# Paving the Way to Eco-Friendly IoT Antennas: Tencel-Based Ultra-Thin Compact Monopole and Its Applications to ZigBee

**DOI:** 10.3390/s20133658

**Published:** 2020-06-30

**Authors:** María Elena de Cos Gómez, Humberto Fernández Álvarez, Alicia Flórez Berdasco, Fernando Las-Heras Andrés

**Affiliations:** TSC. Electrical Engineering Dept., University of Oviedo, 33203 Gijón, Spain; uo194342@uniovi.es (H.F.Á.); uo237201@uniovi.es (A.F.B.); flasheras@uniovi.es (F.L.-H.A.)

**Keywords:** textile antenna, sustainable fabrics, ecofriendly antenna, green antenna, flexible antenna, compact antenna, antenna for IoT, ZigBee, wearable antenna, energy efficient antenna

## Abstract

An ultrathin, compact ecofriendly antenna suitable for IoT applications around 2.45 GHz is achieved as a result of exploring the use of Tencel fabric for the antenna’s design. The botanical ecofriendly Tencel is electromagnetically characterized, in terms of relative dielectric permittivity and loss tangent, in the target IoT frequency band. To explore the suitability of the Tencel, a comparison is conducted with conventionally used RO3003, with similar relative dielectric permittivity, regarding the antenna dimensions and performance. In addition, the antenna robustness under bent conditions is also analyzed by measurement. To assess the relevance of this contribution, the ultrathin ecofriendly Tencel-based antenna is compared with recently published antennas for IoT in the same band and also, with commercial half-wave dipole by performing a range test on a ZigBee-based IoT testbed.

## 1. Introduction

The Internet of Things (IoT), which currently is the preponderant technology trend, could be defined as a platform grouping and interconnection of devices and objects through a network (either private or the Internet), where all of them can be visible and interact, collaborate and exchange data with each other without the need for human intervention. The type of objects or devices could be any, from sensors and mechanical devices to everyday objects such as the fridge, footwear or clothing. 

IoT is already improving our quality of life and the competitiveness of companies, although its benefits will be more noticeable from now on. People’s way of life and doing business will basically change as IoT devices improve their efficiency, lower operating cost and manage expanding markets, despite the pending challenges concerning properly defining use cases and security issues.

Although IoT has countless specific applications, they can be classified by scope or main areas of use: in everyday life, in healthcare, in smart cities, in agriculture, in industrial automation, in retail and in disaster management. The whole communication framework of IoT comprises short range, medium range, and public networks. Some may play a more prominent role than others depending on the specific application. As a result, there are different technologies that impact the IoT, since they are required to connect and control the devices and to transport the generated information. Among the most notable wireless technologies for IoT [1] are: Zigbee, 6LowPAN, LoRa, Z-Wave, WirelessHAR, Bluetooth, Wi-Fi, LTE, NB-IoT, Near-field Communications (NFC) and Radiofrequency Identification (RFID).

Low power consumption is a mandatory requirement for IoT devices. The integration of the RF front end and the control unit into a chip contributes to its achievement, but the antenna is left out in most cases. This adds yet another challenge to those already faced by IoT antenna designers. In addition to the performance, size, cost and ecofriendliness requirement, the antenna has to be highly energy efficient (which is even more challenging if size reduction is simultaneously pursued). Furthermore, for wearable devices ergonomics is another key aspect to consider. All of these requirements are not generally satisfied at once by the currently available IoT antennas, which means that there are still wide open research areas.

The aforementioned wireless technologies involved in IoT work in frequency bands below 6 GHz, which explains the many research works focused on achieving suitable antenna designs at these frequencies, both on rigid and flexible materials, the latter being preferable for wearable devices. In fact, most of these wireless standards operate in the industrial, scientific and medical (ISM) band around 2.45 GHz, with the advantage of lower loses compared to higher frequency bands and profiting from both the channel propagation knowledge and the developed electronics. Zigbee, which is one of the world’s most trusted standards thanks to its interoperability, simplicity of end-user experience, security, stability and reliability, can be used worldwide for IoT at this frequency band.

Different materials have been used to achieve flexible antennas: plastics [2], polydimethylsiloxane (PDMS) [3,4], paper [5], textiles [6,7,8,9,10,11,12], and recently, even a new ceramic material was successfully tested [13]. In addition, some works using substrate-integrated waveguide (SIW) technology [14], mainly at 5.5 GHz, exhibited good performance. However, its inherent requirement of via holes, whose robustness with use can be compromised, is a drawback for wearable devices. All of the aforementioned materials cannot be considered ecofriendly, even if great efforts are made to recycle them. Unfortunately, the manufacturing processes of these materials and the recycling itself still have a major impact on the environment, whether due to water consumption or the generation of waste and pollution. It is well known that there must be a paradigm change that leads to circular economies if environmental damage is to be reduced.

For wearable applications, textile antennas are preferable for the sake of skin comfort. The sustainability and ecofriendliness of the clothing fabric hopefully concerns an increasing number of people, not only in terms of environmental care but also for their own health. However, it is noteworthy that achieving compact antennas on textiles is a challenge, since the relative dielectric permittivity values, ranging from 1.17 for fleece to 2.95 for leather [12], are not high compared to other materials commonly used to that aim.

In this contribution, the possibilities are explored for using a totally ecological textile, not yet used for electronics, as a substrate for an IoT antenna design operating in the 2.4 GHz band. To this aim, it is firstly required to conduct the electromagnetic characterization of the textile at the intended frequency band. This allows us to elucidate if it enables a compact antenna design with suitable performance for an IoT application, in addition to reducing the environmental footprint. For comparison, a commonly used commercial substrate with similar relative dielectric permittivity is also used in the antenna design. The application of the resulting ecofriendly antenna on the novel textile for a ZigBee-based IoT platform is tested, comparing its performance with a typical commercial antenna.

The paper organization is as follows: first, the two substrates considered for the antenna design and fabrication are presented along with their main characteristics. The antenna design is then carried out and its performance described, based on electromagnetic simulation results, including the added value of a comparison with respect to the state of the art on IoT antennas at 2.4 GHz. The operation of the fabricated prototypes on both substrates is compared next, including the behavior under bending conditions for the one based on the ecofriendly textile. The textile-based antenna prototype is then subjected to a range test on a ZigBee-based IoT platform, comparing its performance with the typical half-wave dipole commercial antenna. Finally, some conclusions are drawn.

## 2. Substrates Considered for the Design of the Antenna

This section describes the two substrates that are considered for the design of the antenna: a widely used in electronics commercial substrate, Rogers’ RO3003, which can be used in wearable devices due to its softness and certain degree of conformability, and a totally ecological botanic textile, Tencel fabric, which has not been used previously as an electronics substrate. In consequence, the Tencel fabric has to be electromagnetically characterized prior to the antenna design. As it will be shown, both substrates have similar relative dielectric permittivity, which makes it possible to fairly compare the antenna designs.

### 2.1. Widely Used Commercial Substrate: RO3003

**Figure 1:**
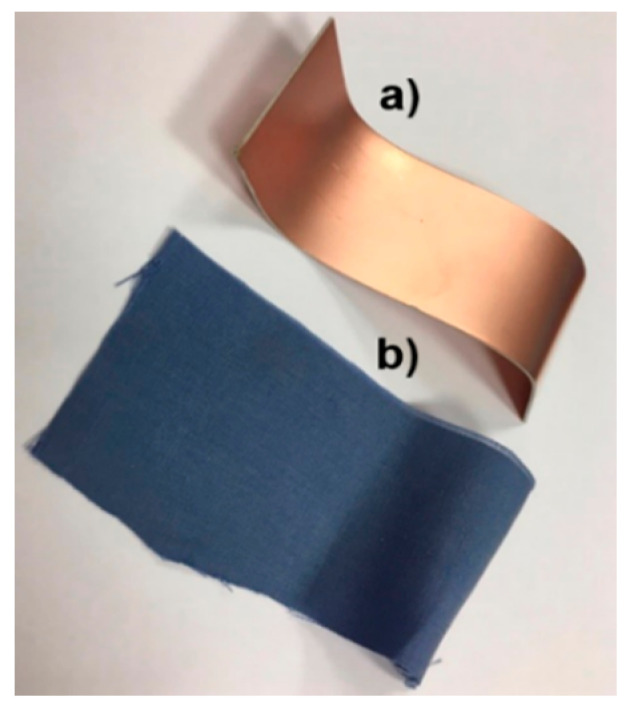
Substrates for the compact coplanar waveguide CPW-fed monopole: (**a**) RO3003 and (**b**) Tencel fabric.

The commercial RO3003 [15] has good electrical and mechanical properties, as well as a competitive price for microwave and RF application, which makes it a material used extensively for electronics. It is made of ceramic-filled PTFE (Teflon) composites that endow it with very stable relative dielectric permittivity with temperature and very low loss tangent. Both are advantages. Specifically, the relative dielectric permittivity is ε_r_ = 3.0 and the loss tangent is tanδ = 0.0013. It cannot be rated as flexible, but it can be considered conformable to some extent (see Figure 1).

Although RO3003 complies with Restriction of Hazardous Substances (RoHS) regulation [16] because its process is free of heavy metals (such as lead, mercury and cadmium) it cannot, however, be considered ecofriendly currently since it is PTFE-based (as are many other commercial substrates).

### 2.2. Botanic Ecofriendly Textile: Tencel

Tencel fabric [17] is obtained from a natural cellulosic fiber of botanic origin, Lyocell, which is man made from the pulp of sustainably farmed trees. The fiber production itself is extremely ecofriendly due to a closed loop system. 

**Figure 2:**
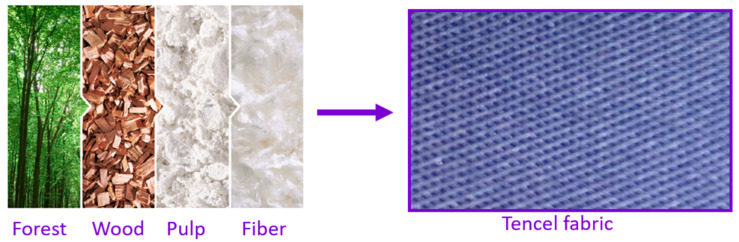
Stages in obtaining the Tencel fabric and a close-up view of a piece of Tencel.

Concerning the environmental footprint, the Tencel fabric (see Figure 2) is ecological due to its ecoresponsible production and it can be reused over and over again, which makes it sustainable, since waste disposal is reduced by 98%. In addition, the Tencel fabric exhibits a series of characteristics that make it especially suitable for implementing wearable devices: it is light and soft, and so is perfect for making garments; it is hygienic, showing higher capabilities to reduce the growth of bacteria without chemical additives that are used in other fabrics; it is breathable, facilitating air circulation and reducing moisture appearance, and lastly, it is antistatic and comfortable to the skin, not irritating polyester or wool usually are.

Tencel has not been used before for electronics. Therefore, it must be electromagnetically characterized. Preliminary studies using a Keysight Technologies 8,5072A 10 GHz split-cylinder resonator yielded a relative dielectric permittivity value ε_r_ = 3.5 at 10 GHz for a sample with thickness h = 0.26 mm. However, it is important to take into account that this characterization is not very accurate due to the low thickness of the sample, which causes a small disturbance of the inherent cylinder resonance, and this disturbance is the base of the characterization. In fact, the cavity fabricator currently recommends using samples in the 0.1–3 mm range, and typically 1 mm. Further attempts were made to improve the accuracy of the characterization by folding the textile to increase the thickness of the sample and by tightening it to prevent air from entering. As a result, for a sample with thickness h = 0.94 mm, a relative dielectric permittivity value of ε_r_ = 2.74 at 10 GHz was obtained. Concerning the loss tangent, tanδ = 0.01 was obtained as an average of the measurements for the two thicknesses. Moreover, the split-cylinder resonator is intended to be used in the characterization of rigid substrates, and so it is not the best solution for a textile, although it gives an idea of the relative permittivity value.

For the intended application of Tencel in antenna design, a characterization method involving transmission lines (which are actually used for the antenna feeding or shaping) at the specific operating frequency would be more reliable, since it will count on the underetching or overetching, depending on the fabrication method and the dielectric mechanical and thermal properties. These deviations in the resulting transmission line thickness lead to frequency shifts in practice, which are not considered in other characterization methods that are based on measuring a pure dielectric sample.

Taking this into consideration, the characterization at the intended 2.4 GHz frequency band was conducted using a microstrip line and a T-resonator [18] on Tencel with a thickness of h = 0.26 mm. The T-resonator is a line of identical characteristic impedance with a λ_g_/4 stub at the intended characterization frequency. These structures are fabricated and subsequently their S21 parameter is simulated using HFSS, tuning ε_r_ and tanδ in the simulation to match the measurement results. The use of this method yielded ε_r_ = 2.36 and tanδ = 0.01 at 2.59 GHz. This value for the relative dielectric permittivity is consistent with the cellulose (wood pulp) composition of the Tencel, as in the case of paper, with a relative permittivity in the range 2–4.

## 3. Compact CPW-Fed Monopole Design

In this section, the design of the compact antenna is explained, starting with the selected feeding technique, the proposed geometry and the resulting dimensions for optimal operation, obtained through simulation for the two substrates. Then, the operating mechanism is described, supported by the surface current distribution. The matching and the radiation characteristics of the antenna on both substrates are shown and compared. Finally, the behavior of the ultrathin compact flexible antenna on ecofriendly textile under bending conditions is analyzed.

### 3.1. Antenna Geometry and Optimized Dimensions 

For the feeding of the antenna, coplanar waveguide (CPW) line is chosen since it provides wide bandwidth while it requires metalizing only one layer, which additionally makes it cheaper and easier to fabricate than microstrip. The reference impedance is 50 Ω. To obtain the feeding line dimensions, with a width W*_L_* and gap g, not only the reference impedance must be considered, but also the dimensions of the commercially available connectors, since there are many combinations of W*_L_* and g that would yield 50 Ω for a given relative permittivity and thickness; however, some could result in short circuits when soldering the connector, depending on the insulating distance between the probe feed and the ground.

**Figure 3:**
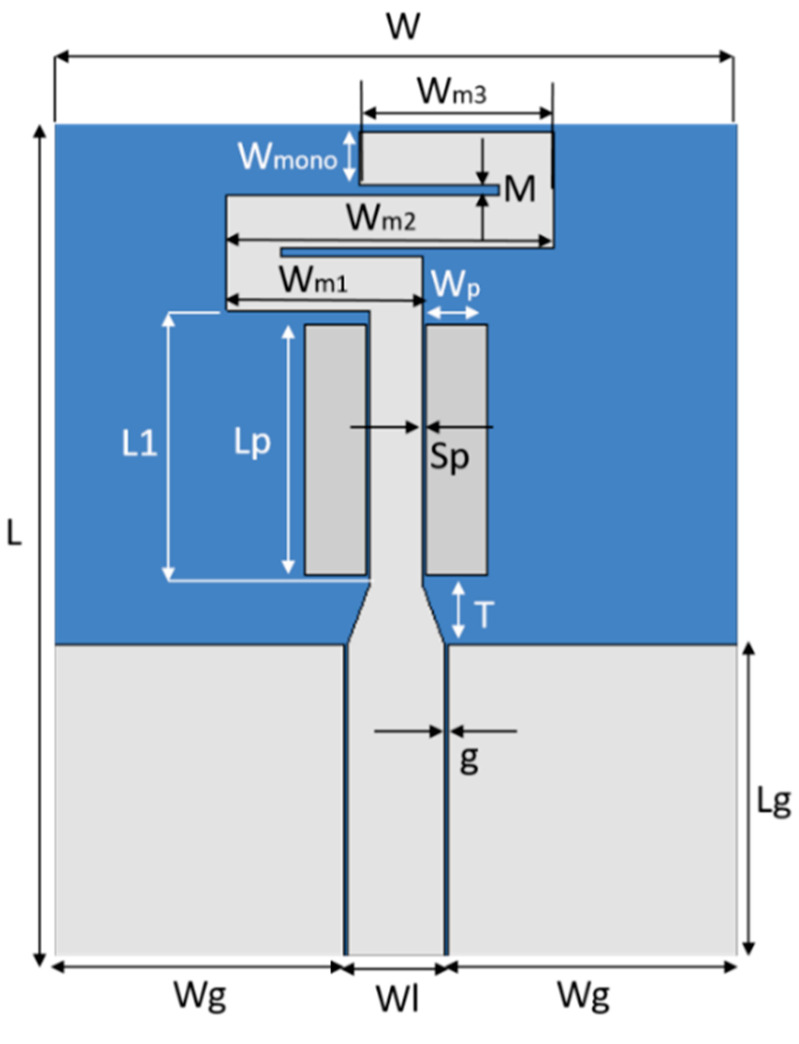
Geometry of the compact CPW-fed monopole antenna.

The starting point for the antenna design is a simple CPW-fed monopole with a length of approximately λ/4 at the intended frequency (31 mm at 2.4 GHz) plus the feeding line length (about 13–14 mm, the same as the ground plane). This would result in an antenna at least 44 mm long. The antennas’ width would depend on the ground-plane width required for a good matching level and bandwidth. The target is to achieve a very compact antenna on both RO3003 and Tencel, with proper impedance matching at the IoT frequency band from 2.4 to 2.7 GHz. To miniaturize the antenna, the strip corresponding to the monopole is narrowed and partially meandered from its upper end, and a tapering introduced on its bottom end to improve the impedance matching to one of the feeding lines. In addition, two parasitic strips are arranged at both sides of the nonmeandered strip for further bandwidth broadening (see Figure 3).

A parametric analysis was conducted using finite element method FEM-based 3D electromagnetic simulation using HFSS commercial software, to optimize the antenna design at the intended frequency band of operation for IoT. The center frequency is mainly controlled by Wm3, Wm2, Wm1 and L1 (as they increase the frequency shifts down) and by the gap between meanders M (as it increases, the frequency decreases). The bandwidth is enlarged and the matching level improved as the gap Sp between the two side parasitic rectangles and the strip decreases. The strip tapering length T influences both the center frequency (goes down as T increases) and the bandwidth (slightly decreases as T increases). Finally, concerning the ground plane dimensions, increasing the length Lg barely decreases the frequency once a sufficiently large value is adopted. Broadening the width Wg shifts down the operating band but also worsens the reflection coefficient, although only slightly. Table 1 indicates the optimized antenna dimensions for operation from 2.4 to 2.7 GHz using both RO3003 and Tencel.

According to Table 1, the size in terms of area (L × W) of the optimized CPW-fed slot monopole antenna based on both RO3003 (28.4 × 34.5 mm^2^) and Tencel (28.3 × 34.5 mm^2^) is almost identical. However, it is very important to highlight that the antenna design on the ecofriendly Tencel is three times thinner than the one in RO3003 and, in addition, is totally flexible and skin comfortable. Moreover, the initial CPW-fed monopole on both substrates, with identical ground plane, would be much longer (L = 45.5 mm, at least) so that the compact design leads to a 24% size reduction.

### 3.2. Antenna Matching

**Figure 4:**
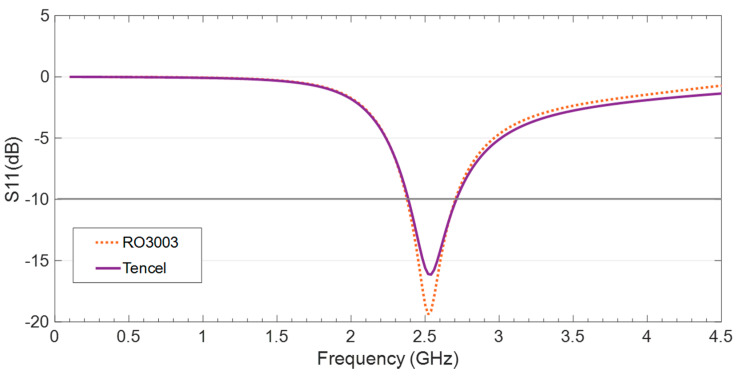
Reflection coefficient results, S11(dB), obtained in the simulation for the compact CPW-fed monopole on RO3003 and Tencel.

The reflection coefficient for the CPW-fed monopole on both RO3003 and Tencel with the optimized dimensions is obtained in the simulation and the results depicted in Figure 4. Observe that the impedance matching achieved is suitable throughout the intended IoT frequency band around 2.45 GHz.

The specific operation frequencies as well as the corresponding bandwidths obtained in the simulation for the antenna on the two substrates are included in Table 2. It is worth noting that the Tencel makes it possible to achieve the same bandwidth at the target frequencies as the RO3003, with the advantages of being three times thinner and ecofriendly.

**Figure 5:**
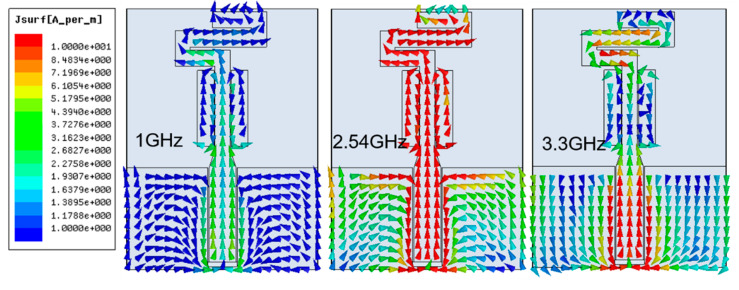
Surface current distribution for the compact CPW-fed slot monopole at the center frequency of the operative band (2.54 GHz) and at two frequencies (1 and 3.3 GHz) outside the operative band (without proper impedance matching).

Figure 5 shows the surface current distribution of the antenna on Tencel at three different frequencies: two nonoperative ones (1 and 3.3 GHz) and one at the center of the operative band (2.54 GHz). At 2.54 GHz, high levels of current can be observed both in the meandering strip and in the straight section of the strip, as well as in the side parasitic rectangles, in accordance with the behavior described for the radiating structure and observed in the parametric analysis. Of course, there are also high currents near the feeding line. As could be expected, at 1 and 3.3 GHz the currents’ level on the antenna is low since the antenna is not operative.

### 3.3. Radiation Properties of the Antenna

The radiation properties of the CPW-fed monopole were analyzed in the simulation for the two substrates under consideration. The results obtained concerning the peak-realized gain G (dB), the directivity D (dB) and the radiation efficiency η (%), for three frequencies within the intended 2.4–2.7 GHz band are shown in Table 3.

As it can be observed, the CPW-fed monopole antenna on both RO3003 and Tencel provides almost identical radiation properties that are suitable for IoT applications. Once more, it is remarkable that the Tencel is three times thinner, ecofriendly and preferable for wearable applications. In addition, it is important to highlight that the radiation efficiency is the key parameter involved in ensuring low power consumption, and the concerned results are excellent and even more relevant considering how challenging is reducing size while preserving high radiation efficiency.

**Figure 6:**
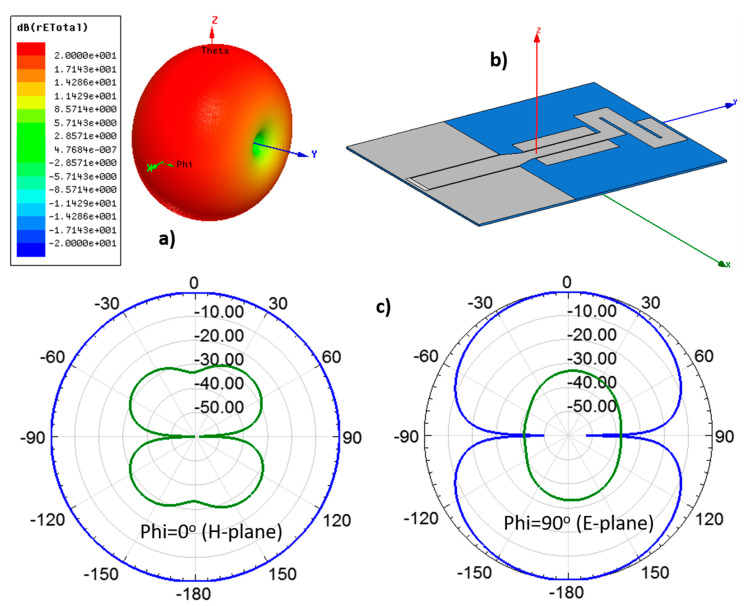
Radiation pattern obtained in the simulation for the compact CPW-fed monopole on Tencel at 2.54 GHz: (**a**) Three-dimensional pattern, (**b**) Antenna arrangement to simulate its radiation properties and (**c**) radiation pattern cuts for Phi = 0° and Phi = 90°. The blue traces stand for copolarization (CP) and the green ones for cross-polarization (XP).

Figure 6 shows the radiation patterns of antenna on Tencel at the center frequency of the operating band, 2.54 GHz, including the 3D view and the cuts for Phi = 0° (H-plane) and Phi = 90°(E-plane). The expected monopole-like radiation pattern, omnidirectional for the H-plane, can be observed.

## 4. Comparison with the State-of-the-Art on IoT Antennas

To endorse the achievements of this work, a comparison with recently published antenna designs working at the 2.45 GHz band in terms of size, relative dielectric permittivity, bandwidth and radiation efficiency is required. The data for this comparison are included in Table 4.

At a glance, it can be observed that the compact CPW-fed monopole on the ecofriendly Tencel is much smaller in size than most of the recent antenna designs, including those on substrates with higher relative dielectric permittivity [19], while providing much wider bandwidth (even than those on thicker substrates with lower εr [20,21,22,23]) and higher radiation efficiency, which is a key fact for ensuring low power consumption in IoT devices. An antenna slightly smaller than the one presented in this work is shown in [22], but it suffers from the disadvantages of providing lower radiation efficiency and being more than twice as thick. Moreover, in literature one can find improved designs in terms of bandwidth and isolation from the body, based on adding an Electromagnetic Band-gap EBG (as in [24], which enhances the design of [22]) but at the expense of increasing the antenna profile. In any case, this type of technique could also be considered for the antenna of this work. Therefore, the presented antenna goes beyond the state-of-the-art in overcoming previous designs, while it paves the way to ecofriendly IoT antennas.

## 5. Fabricated Prototypes of Compact Wearable Antenna

**Figure 7:**
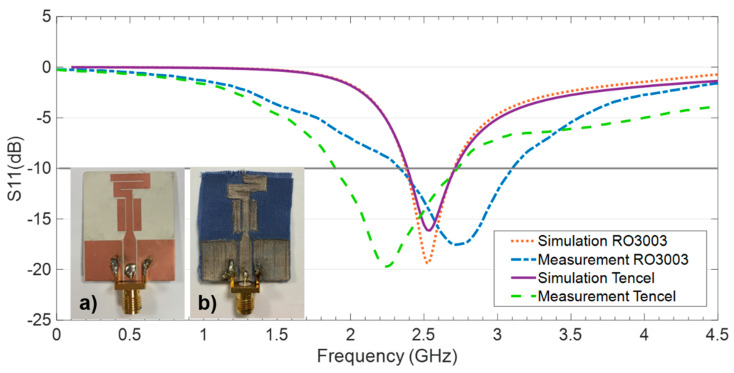
Fabricated prototypes of the CPW-fed monopole antenna on (**a**) RO3003 and (**b**) Tencel. The results for the reflection coefficient, S11(dB), in the simulation and measurement for the prototypes are shown.

The CPW-fed monopole was fabricated using the two substrates under consideration: the conventionally used RO3003 and the botanic Tencel proposed as a novel dielectric to be used for achieving ecofriendly antennas. The fabricated prototypes can be observed in Figure 7.

The RO3003 comes copper plated from the factory and therefore the metallic parts of the antenna on RO3003 are made of copper. With the Tencel, Shieldit Super electrotextile was used, which incorporates a hot melt adhesive on its back to fix it to the Tencel; it is RoHS compliant and exhibits low corrosion.

### 5.1. Antenna Prototypes: On Tencel vs. on RO3003

Figure 7 shows the reflection coefficient results obtained in the simulation and measurement for the fabricated prototypes on both RO3003 and Tencel. It is important to note that the connector was not included in the simulation and that they are hand-soldered close to the feeding gap, which brings about a similar disturbance in both prototypes in terms of slightly shifting (upwards for RO3003 and downwards for Tencel) and broadening of the frequency band. Nonetheless, suitable impedance matching levels were achieved in measurements made in the target IoT frequency band, from 2.4 to 2.7 GHz, for the prototypes on both RO3003 and Tencel.

### 5.2. Operation under Bending Condition of the Tencel-Based Ultrathin Compact Monopole

**Figure 8:**
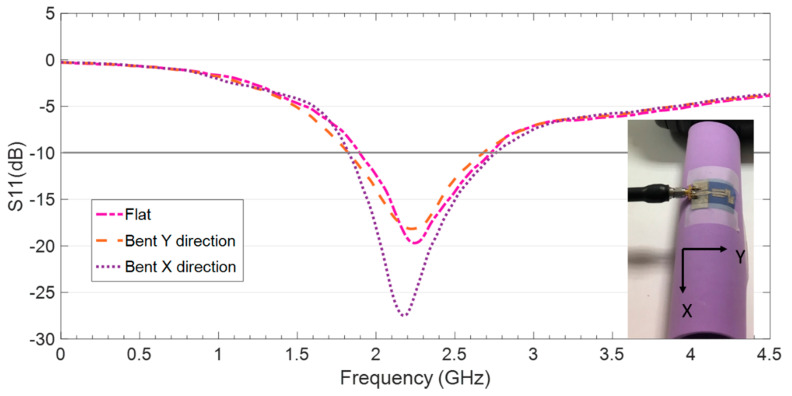
Reflection coefficient results for the compact CPW-fed monopole on Tencel, measured under flat and bent conditions using a foam cylinder of radius R = 25 mm.

A relevant issue in wearable antennas is their robustness in eventual bending situations. Although Tencel fabric is not easily wrinkled at all, due the smoothness, elasticity and high wrinkle resistance of the Lyocell fibers that compose it, it is interesting to study how the antenna behaves when it is bent in different directions. A foam cylinder of radius R = 25 mm was used to bent the antenna in both X and Y directions. The reflection coefficient of the antenna on Tencel was measured under these conditions and the results are shown in Figure 8, along with the ones corresponding to flat layout. It can be observed that the antenna keeps proper impedance matching under both bending patterns. When the antenna is bent in the X-direction, the impedance matching level improves and the bandwidth slightly increases. Therefore, it can be asserted that the antenna is robust in terms of bending, even if the impedance matching slightly worsens when it is bent in the Y direction.

## 6. Tencel-Based Monopole Operation on a ZigBee-Based IoT Platform

Many aspects can influence the quality of the wireless communication between XBee modules: the type of antenna and arrangement, absorption, line-of-sight conditions, reflection of waves etc. To study the link quality and the RF range in real-world conditions for two XBee modules in the same network, a range test can be conducted [25]. The XCTU software can perform a range test for at least one local node (XBee module connected to a computer) and a remote node, both in the same network. The local node sends packets to the remote one and waits for the echo from the remote node. Meanwhile, the number of packets sent and received by the local node and the received signal strength indicator value (RSSI) of both sides are counted and measured by the software, respectively. To ensure that every packet sent from the local node is received as an echo by the same node, transparent mode needs to be configured in the remote node and its loopback jumper should be closed, while the local node has to be configured in the application programming interface (API) mode to enable reading the remote node RSSI level. The data retrieved during the range test are provided by the XCTU (see Figure 10) in three formats: RSSI chart (values of the nodes along the test, as well as percentage of the sent packet success), local and remote instant RSSI (for the last packet sent/received) and packet summary (total sent, received and lost, and transmission errors, together with the percentage of successfully sent and received packets throughout the test).

**Figure 9:**
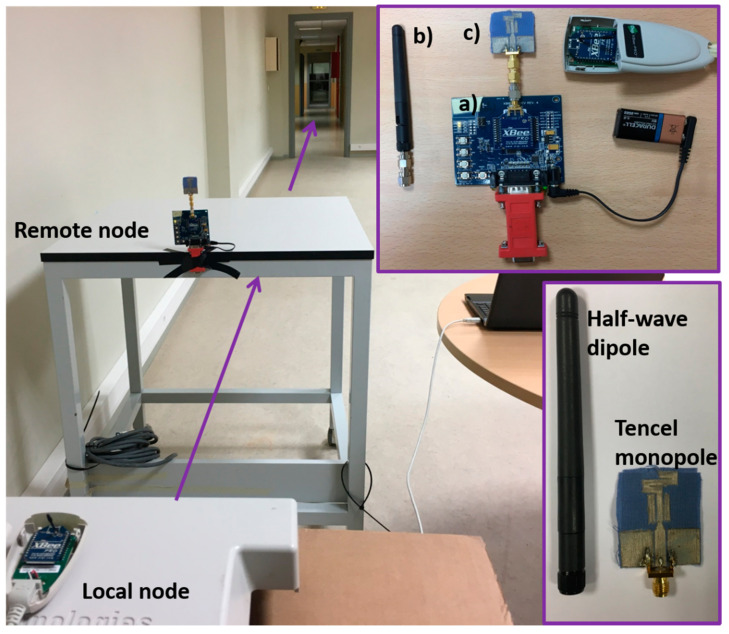
Setup for the range test on a ZigBee-based IoT platform: (**a**) XBee Pro module with loopback, (**b**) half-wave dipole and (**c**) Tencel-based monopole.

Accordingly, the setup (see Figure 9), used to test the novel ecofriendly textile antenna and compare it to a typical commercial half-wave dipole, involves two XBee Pro modules: a fixed local node connected to a laptop (using a whip antenna with 1.5 dBi gain) and a mobile remote node (using either A24-HASM-450 half-wave dipole with 2.1 dBi gain or Tencel monopole) with loopback that sends back any received packet to the local node. The XBee Pro modules were set to minimum power level. Zigbee and 802.15.4 standard break the 2.4 GHz band into 16 channels, from channel 11 (0 × 0B) at 2.405 GHz to channel 26 (0 × 1A) at 2.480 GHz. Several channels were used for the range test and the retrieved data at 24.5 m distance are indicated in Table 5. 

**Figure 10:**
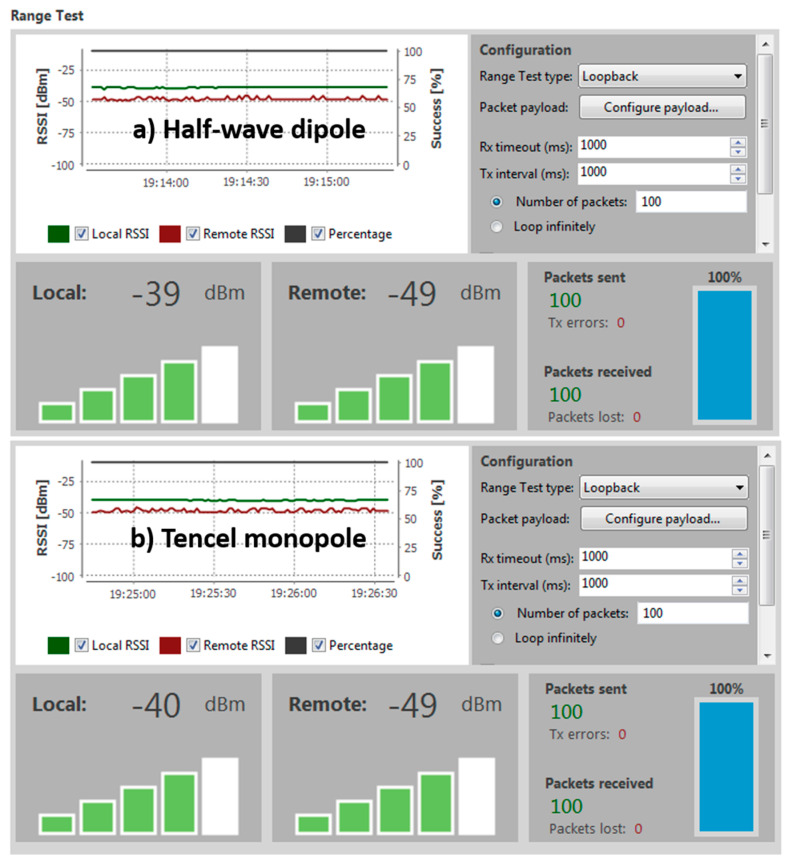
Results of the range test on the ZigBee-based IoT platform using the 0 × 11 channel obtained with the XCTU software.

During the range test carried out on three different channels, 100% of the packets were both correctly sent and received. It is remarkable that for channel 0 × 11 at 2.435 GHz, the RSSI and the percentage of packets successfully received were almost identical for both antennas (see Figure 10). However, it is important to highlight that the optimized Tencel-based monopole is much smaller than the dipole, as well as lighter and easily wearable. Therefore, the antenna on Tencel works perfectly for this IoT technology, while being ultrathin, compact and ecofriendly.

## 7. Conclusions

The use of a botanical textile, Tencel, as an antenna substrate was explored with success. As a result, an ecofriendly, compact and ultrathin flexible monopole was achieved and its radiation properties found suitable for wearable IoT devices operating in the 2.4 GHz band.

The comparison of the Tencel with the RO3003, which is a commercial substrate widely used in electronics, yielded remarkable conclusions. The radiation efficiency of the Tencel-based antenna is preserved, compared to the RO3003-based one, while the Tencel is much thinner, flexible and skin friendly. Furthermore, Tencel costs much less than the RO3003.

Regarding other textiles used up to now in antennas, the Tencel makes it possible to achieve ecofriendly and more compact designs, since it combines the advantages of being a fully recyclable material and having a higher relative dielectric permittivity (similar to that of paper, since it is also composed of cellulose, but being much more robust than it).

Comparison with the state-of-the-art on IoT antennas shows that the Tencel-based monopole presented is much more compact and thinner than recent wearable antennas at 2.45 GHz, while providing wider bandwidth and radiation efficiency. 

When the performance of the Tencel-based monopole is compared to that of a typical commercial half-wave dipole in a Zigbee-based range test, almost identical results are obtained. This indicates that the Tencel-based antenna is suitable for IoT in this frequency band. However, it should be noted that the Tencel-based antenna is much smaller than the dipole and is also ultrathin and ecofriendly, which makes it advantageous. 

## Figures and Tables

**Table 1:** Dimensions of the CPW-fed monopole antenna for different dielectric substrates.

Substrate	Dimensions (mm)								
	L	W	h	W_g_	L_g_	W_L_	g	L_L_	W_p_
RO3003	34.5	28.4	0.76	12	13.5	4	0.20	11	2.5
Tencel	34.5	28.3	0.26	12	13.5	4	0.15	11.4	2.5
	**W_mono_**	**W_m1_**	**W_m2_**	**W_m3_**	**M**	**T**	**L_p_**	**S_p_**	
RO3003	2.2	7.1	11.5	12.4	0.28	2.39	10	0.1	
Tencel	2.2	8.1	13.5	8	0.38	2.39	10.4	0.15	

**Table 2:** Frequency bands and bandwidths of the compact CPW-fed monopole on different substrates.

Substrate	Lower Band			
	Freq (GHz)		BW	
	f_Low_	f_Up_	Total (MHz)	%
RO3003	2.377	2.705	328	13
Tencel	2.387	2.710	323	13

**Table 3:** Radiation properties of the CPW-fed monopole antenna obtained for different dielectric substrates in simulation.

Freq. (GHz)	RO3003			Tencel		
	G (dB)	D (dB)	η (%)	G (dB)	D (dB)	η (%)
2.40	1.93	2.23	93	1.86	2.23	92
2.54	2.24	2.25	100	2.17	2.25	98
2.70	1.88	2.26	92	1.87	2..26	92

**Table 4:** Data for performance comparison of the proposed antenna with previous antenna designs in terms of size, relative dielectric permittivity, bandwidth and radiation efficiency.

References	Dimensions in mm^3^	ε_r_	BW (%)	η (%)
[19]	56 × 65.5 × 1	4.4	5.09	73
[21]	63 × 24.8 × 7.3	2.2	4	-
[22]	30 × 20 × 0.7	1.7	15	79
[23]	59.6 × 59.6 × 3.7	1.5	4.8	81
[20]	46 × 25 × 2	1.2	3.41	-
This work	28.3 × 34.5 × 0.26	2.36	13	92

**Table 5:** RSSI measured results on different frequency channels during the range test for the local and remote ZigBee nodes using the commercial half-wave antenna and the Tencel monopole.

Channel Hex	Freq. (GHz)	Antenna	Local RSSI (dBm)	Remote RSSI (dBm)
0 × 0C	2.410	Half-wave dipole	−37	−42
		Tencel monopole	−40	−44
0 × 11	2.435	Half-wave dipole	−39	−49
		Tencel monopole	−40	−49
0 × 17	2.465	Half-wave dipole	−50	−57
		Tencel monopole	−49	−54
